# The dose-dependent efficacy of esketamine in spinal surgery with intraoperative neuroelectrophysiological monitoring: a randomized controlled trial

**DOI:** 10.3389/fmed.2025.1579908

**Published:** 2025-06-18

**Authors:** Chunyan Lin, Jianlin Wang, Long Zhang, Liyong Yuan, Guanyi Liu, Miao Zhu, Liangguang Zhang

**Affiliations:** ^1^Department of Anesthesiology, Ningbo No. 6 Hospital, Ningbo, Zhejiang, China; ^2^Ningbo Clinical Research Center for Orthopedics, Sports Medicine & Rehabilitation, Ningbo, Zhejiang, China; ^3^Spine Surgery Center, Ningbo No. 6 Hospital, Ningbo, Zhejiang, China

**Keywords:** esketamine, clinical efficacy, dose, spinal surgery, intraoperative neuroelectrophysiological monitoring

## Abstract

**Purpose:**

This study aims to validate the efficacy and safety of combining different doses of esketamine with propofol, remifentanil, and dexmedetomidine in spinal surgery under intra-operative neuroelectrophysiological monitoring (IONM).

**Methods:**

All enrolled patients underwent a total intravenous anesthesia (TIVA) maintenance regimen, which included propofol, remifentanil, and dexmedetomidine. The patients were randomly assigned to four groups based on the use and dosage of esketamine: Group Control (TIVA + NS), Group A (TIVA + Esketamine 0.1 mg/kg/h), Group B (TIVA + Esketamine 0.3 mg/kg/h), and Group C (TIVA + Esketamine 0.5 mg/kg/h). The study measured vital signs, consumption of anesthetics, operation time, blood loss, awakening time in the postanesthesia care unit (PACU), visual analog scale (VAS) pain score, quality of recovery (QoR) -15 score, and dosage of supplementary analgesics. Additionally, adverse postoperative reactions were recorded.

**Results:**

Group B had lower dosages of propofol (*P* = 0.021), remifentanil (*P* = 0.001), and dexmedetomidine (*P* < 0.001) than the Control Group, while Group C had lower dosages of remifentanil and dexmedetomidine (*P* < 0.001) than the Control Group. The postoperative mean arterial pressure (MAP) was lower in Group B than in the Control Group (*P* = 0.028). Patients in Group C experienced a prolonged awakening time (*P* < 0.001) but had lower VAS pain scores at PACU than those in the Control group (*P* = 0.044). Both QoR-15 scores and MoCA scores were significantly higher for patients in Groups A, B, and C compared to those of the Control group (QoR-15: *P* = 0.001, < 0.001, < 0.001; MoCA: *P* = 0.004, < 0.001, < 0.001). Group B had few postoperative complications.

**Conclusion:**

The dose of 0.3 mg/kg/h esketamine is safe and effective for spinal surgery with IONM, improving control of postoperative complications.

## Introduction

Intraoperative neuroelectrophysiological monitoring (IONM) is widely used and employed in major spinal surgery (1, 2). IONM ensures patient safety and surgical efficacy during major spinal procedures. By enabling real-time monitoring and recording of the patient’s neurological status, it aids doctors in timely detection and prevention of potential nerve damage, enhances their understanding of the operative process, and facilitates adjustment of treatment plans based on actual circumstances (1, 3).

An appropriate anesthesia protocol is crucial for ensuring the accuracy of IONM. Currently, the most commonly employed regimen is total intravenous anesthesia (TIVA), combining propofol, remifentanil, and dexmedetomidine (4). However, the high dosage administration of these drugs may still impact potential amplitude, leading to signal attenuation or distortion and consequently reducing the precision of IONM (5). Furthermore, due to interindividual variations in drug response, the stability of the circulatory system can be compromised during surgery, resulting in significant fluctuations in heart rate and blood pressure that increase perioperative risks (6, 7).

Ketamine is a potent general anesthetic with analgesic solid effects, sympathetic excitation and inhibition of hyperalgesia (8). Previous studies have demonstrated that the combination of ketamine and propofol exhibits complementary effects (9). Esketamine, the destroy isomer of ketamine, not only retains its original characteristics, such as sympathetic excitation, mild respiratory depression, and potent analgesia but also mitigates psychiatric adverse reactions (10, 11). Meanwhile, esketamine contributes to enhanced recovery after surgery (ERAS) and improves the controllability (10, 11). However, more research is needed on using esketamine in spinal surgery under IONM.

The primary objective of this study is to evaluate the efficacy and safety of incorporating esketamine into the standard TIVA regimen for spinal surgery under IONM. Additionally, the study seeks to explore potential dose-dependent effects of esketamine on the circulatory stability and perioperative safety. The research aims to provide evidence-based insights into optimizing anesthesia protocols for spinal surgeries requiring IONM, ultimately contributing to improved patient safety and surgical efficacy.

## Materials and methods

This study is a prospective, double-blinded, randomized controlled trial. Prior to the commencement of the study, the institution conducted an ethical review and obtained ethical approval (L2021075). The trial was registered in the Chinese Clinical Trial Registry on 27 April 2022, under registration number ChiCTR2200059269.

### Patient enrollment

Eligible participants included patients who underwent spinal surgery with intraoperative neurophysiological monitoring (IONM) between 1 May 2023 and 30 April 2024. Informed consent was obtained from all participants who voluntarily agreed to participate in the study. The sample size was determined specifically for the primary outcome (intraoperative propofol consumption) based on pilot data obtained from the Pre-experiment. Using PASS software with a one-way ANOVA design (α = 0.05, power = 0.9, four groups), the initial calculation yielded a minimum required sample size of *n* = 16. A conservative Bonferroni adjustment was applied for planned *post hoc* comparisons. Considering an anticipated attrition rate of 20%, the final sample size was determined to be *N* = 80. Inclusion criteria encompassed the American Society of Anesthesiologists (ASA) classification I-II, body mass index (BMI) ranging from 18.5 to 30 kg/m^2^, and age between 18 and 75 years for both male and female individuals. Exclusion criteria consisted of patients with contraindications or dependence on relevant medications, hyperthyroidism, pheochromocytoma, psychiatric history, the change in surgical methods, the occurrence of serious complications related to surgery and the need for secondary surgery.

### Trial design

In this study, all enrolled patients underwent a TIVA maintenance regimen during surgery, which included the administration of propofol, remifentanil, and dexmedetomidine in combination. The patients were randomly allocated into four groups: Group Control (TIVA + NS), Group A (TIVA + Esketamine 0.1 mg/kg/h), Group B (TIVA + Esketamine 0.3 mg/kg/h), and Group C (TIVA + Esketamine 0.5 mg/kg/h). The randomization process employed a simple digital method, and the allocation was performed by an independent individual not involved in the testing.

### Anesthesia

The patients were strictly fasted for 8 h and prohibited from drinking for 2 h prior to the surgical procedure, with no administration of preoperative medications. Upon entering the operating room, oxygenation was initiated through a mask while peripheral venous access was established. Electrocardiography, pulse oximetry, blood pressure, bispectral index monitoring (BIS), and body temperature were continuously monitored. Anesthesia induction involved the administration of midazolam at a dose of 0.05 mg/kg, propofol at a dose of 2 mg/kg, sufentanil at a dose of 0.3 μg/kg, and rocuronium at a dose of 0.6 mg/kg.

The dosage was monitored and continuously adjusted throughout the operation to maintain appropriate vital signs and BIS. The maintenance dose of propofol was set at 6 mg/kg/h, with incremental adjustments of 1 mg/kg/h as needed. The maintenance dose of remifentanil was established at 0.15 μg/kg/min, allowing for adjustments of 0.05 μg/kg/min each time. For dexmedetomidine, the maintenance dose was 0.5 μg/kg/h, with possible adjustments of 0.1 μg/kg/h until a satisfactory anesthetic effect was achieved. All four groups of patients adhered strictly to this unified anesthesia maintenance protocol.

### IONM

**FIGURE 1:**
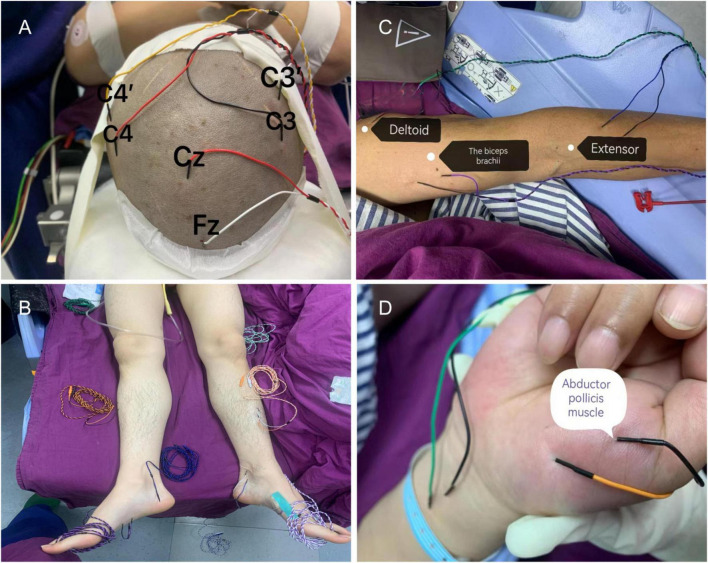
Intraoperative neuroelectrophysiological monitoring probe placement. **(A)** The recording electrodes for somatosensory evoked potential (SEP) are positioned at Cz, Fz, C3’, and C4’, while the stimulation electrodes for motor evoked potential (MEP) are placed at C3 and C4. **(B)** SEP is recorded by stimulating the gastrocnemius muscle to assess sensory function in the lower limb, with the tibialis anterior muscle serving as the recording electrode for MEP. **(C)** SEP is recorded by stimulating both the anterior tibial nerve and median nerve to evaluate sensory function in the upper limb. **(D)** MEP recording electrodes are positioned in both the interossei muscle and flexor pollicis brevis muscle group.

The international 10/20 system standard positioned the monitoring electrode using subcutaneous needle and surface iron electrodes. Somatosensory evoked potential (SEP) employed a constant current single-phase pulse, with tibial nerve stimulation applied to the lower limbs. Recording electrodes were placed at Cz and Fz positions. The median and ulnar nerves were stimulated for upper limb stimulation while recording electrodes were positioned at C3’ Fz and C4’ Fz locations. Stimulation intensity was set at 30 mA with a frequency of 4.1 Hz. Motor-evoked potential (MEP) was induced through transcranial electrical stimulation using a constant voltage electrical stimulator. Stimulation electrodes were placed at C3 and C4 sites while recording electrodes were positioned on bilateral anterior tibial muscles, thenar muscles, and abductor pollicis brevis muscle groups (Figure 1). The stimulation voltage used was 220 V.

The Endeavor CR 16-channel intraoperative monitor (Nicolet, United States) was utilized for the study. TES-MEP monitoring involved placing two disk surface stimulation electrodes at the C’ and C positions, each serving as an anode or cathode. In contrast, the anode acted as the stimulation pole. The contralateral tibialis anterior muscle and plantar flexor digitorum brevis (increased in patients with cervical spondylosis) were recorded for TES-MEP measurements. For CSEP monitoring, stimulation of the posterior tibial nerve at bilateral medial malleolus (increased in patients with ulnar nerve involvement due to cervical spondylosis) was performed, and head CSEP responses were recorded accordingly. Additionally, simultaneous use of the posterior tibial nerve at ankle level and plantar flexor brevis as stimulating nerves and potential recording muscles allowed for a train-of-four twitch test (TOF). During surgery, TES, MEP, and CSEP were monitored sequentially.

### Outcomes

The age, gender, BMI, ASA classification, and surgical methods of the patients enrolled in the study were documented. The primary outcome was the intraoperative consumption of propofol, the key secondary outcome was the intraoperative consumption of remifentanil and dexmedetomidine, and the secondary outcomes were blood pressure and heart rate, awakening time, visual analog scale (VAS) pain score at the postanesthesia care unit (PACU), operation time, blood loss, postoperative delirium score, quality of recovery (QoR) -15 score, and length of stay. Additionally, close attention was paid to postoperative adverse reactions, including stupor vigilans (a clinical state observed during the recovery phase from general anesthesia, wherein, despite the gradual return of consciousness, an individual exhibits persistent muscular rigidity and marked psychomotor inhibition, resembling a stupor state), nausea and vomiting, lethargy, headache, mental disorders, diplopia, hypertension, and heart rate. Neither patients nor observers were only aware of the experimental design and grouping during the data collection process.

### Statistics

Data processing and statistical analysis were conducted using SPSS software (ver.26.0). The normality of continuous data was assessed using the D’Agostino and Pearson test, with normally distributed data presented as Mean ± SD and non-normally distributed data expressed as median (M) with upper and lower quartile ranges. Count data were reported as numbers. One-way ANOVA was used to analyze normally distributed continuous variables, corrected based on results from the Welch variance homogeneity test, and the Bonferroni test was employed for *post hoc* comparisons. Chi-square tests were performed for binary outcomes, whereas the Kruskal-Wallis test compared non-normal distribution or ordinal data between groups. Statistical significance was set at *P* < 0.05.

## Results

**FIGURE 2:**
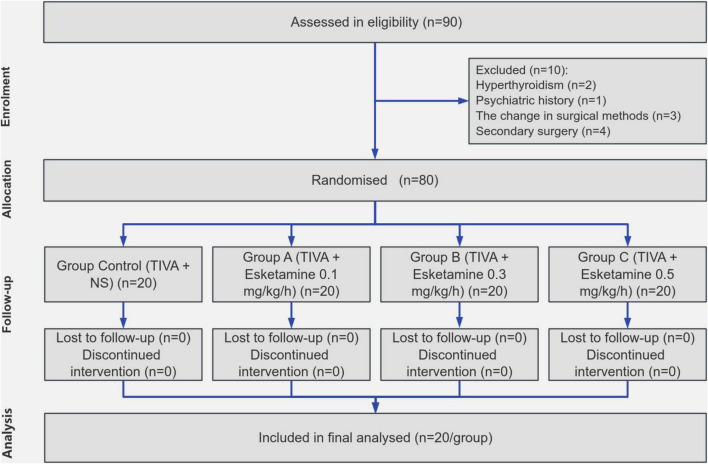
Flow diagram. TIVA, total intravenous anesthesia.

In total, 80 patients were enrolled in the study, no one was excluded from the experiment (Figure 2). All participants underwent the surgical procedure successfully and completed postoperative data collection without any dropouts. No statistically significant differences were observed among the four groups regarding age, BMI, gender, ASA classification, and surgical methods (Table 1).

**TABLE 1:** Patients characteristics (*n* = 20).

Characteristics	Group control	Group A	Group B	Group C	F	*P*
Age (years)	61.1 ± 12.2	57.2 ± 12.6	57.7 ± 12.5	58.9 ± 11.9	0.404	0.750
BMI (kg/m^2^)	24.10 ± 2.81	24.32 ± 2.94	23.12 ± 2.66	24.12 ± 2.46	0.792	0.502
Gender (male/female)	10/10	9/11	11/9	10/10	–	0.940
ASA classification (I/II)	2/18	3/17	3/17	2/18	–	1.000
Surgical methods						
Anterior cervical decompression	9	7	10	9	–	0.945
Posterior cervical decompression	5	9	5	7	–	–
Spinal deformity	2	2	3	2	–	–
Resection of spinal lesions^#^	4	2	2	2	–	–

^#^Tumor or tuberculosis. BMI, body mass index; ASA, American Society of Anesthesiologists.

The results of all observed indicators are presented in Table 2. There were no significant differences in operation time and blood loss among the four groups (*P* = 0.640, 0.843). No significant differences between Group A and Group Control were found in the propofol, remifentanil, and dexmedetomidine dosage (*P* = 1.000, 0.983, 1.000). The dosage of propofol, remifentanil, and dexmedetomidine was significantly lower in Group B than in Group Control (*P* = 0.021, 0.001, < 0.001). The dosage of remifentanil and dexmedetomidine was significantly lower in Group C than in Group Control (*P* < 0.001). No significant difference was found in the dosage of propofol (*P* = 0.239). There were no significant differences among the four groups regarding preoperative MAP and HR (*P* = 0.362, 0.177), nor intraoperative MAP (*P* = 0.898). The postoperative MAP of Group B was lower than that of Group Control (*P* = 0.028).

**TABLE 2:** Observed indices (*n* = 20).

Observed indices	Group control	Group A	Group B	Group C	F	*P*
Operation time (h)	2.35 ± 0.54	2.50 ± 0.54	2.38 ± 0.48	2.53 ± 0.53	0.565	0.640
Blood loss (ml)	185.0 ± 112.5	207.5 ± 116.2	212.5 ± 113.4	195.0 ± 103.7	0.248	0.863
Propofol (mg)	810.8 ± 181.0	773.3 ± 232.3^ns^	620.0 ± 165.4*	678.8 ± 212.8^ns^	3.810	0.013
Remifentanil (μg)	194.8 ± 57.7	179.0 ± 52.1^ns^	129.0 ± 32.4^***^	118.8 ± 29.0^***^	13.580	< 0.001^#^
Dexmedetomidine (μg)	106.0 ± 28.2	104.5 ± 23.9^ns^	67.3 ± 15.1^***^	51.8 ± 11.8^***^	38.879	< 0.001^#^
Esketamine (mg)	0	17.0 ± 4.5	45.1 ± 11.3	85.3 ± 24.4	149.549	< 0.001
Preoperative MBP	103.7 ± 9.1	101.8 ± 7.3	100.1 ± 9.3	104.6 ± 8.5	1.081	0.362
Preoperative HR	65.7 ± 5.5	67.2 ± 6.4	63.6 ± 5.0	64.1 ± 5.8	1.688	0.177
Intraoperative MBP	90.5 ± 10.2	91.6 ± 7.8	90.3 ± 8.2	92.0 ± 7.8	1.103	0.898
Postoperative MBP	104.6 ± 6.9	102.6 ± 5.4^ns^	98.3 ± 8.0*	101.8 ± 7.1^ns^	2.958	0.038
Awakening time (min)	22.4 ± 6.6	21.2 ± 5.6^ns^	20.3 ± 4.6^ns^	33.9 ± 4.3^***^	28.474	< 0.001
VAS pain score at PACU	3.1 ± 0.7	3.0 ± 0.5^ns^	2.9 ± 0.4^ns^	2.6 ± 0.5*	3.057	0.033
QoR-15 score	111.2 ± 3.2	115.3 ± 2.7^**^	124.4 ± 3.8^***^	121.8 ± 2.8^***^	74.639	< 0.001
MoCA score	19.6 ± 1.0	21.1 ± 1.3^**^	23.7 ± 1.5^***^	21.4 ± 1.3^***^	33.055	< 0.001
Length of stay	8.4 ± 1.4	9.3 ± 1.8	8.7 ± 1.4	8.5 ± 1.0	1.377	0.256

Compared to Group Control, ^ns^*P* > 0.05, *P < 0.05, **P < 0.01, ***P < 0.001, ^#^use the Welch test. MAP, mean arterial pressure; HR, heart rate; PACU, postanesthesia care unit; QoR-15, quality of recovery-15; MoCA, montreal cognitive assessment.

The awakening time of patients in Group A and Group B did not differ significantly from that in the Control Group (*P* = 1.000, 1.000). In contrast, patients in Group C experienced a prolonged awakening time (*P* < 0.001). The VAS pain score at the PACU after recovery showed no significant difference between patients in Group A and Group B (*P* = 1.000, 1.000), whereas patients in Group C had lower VAS pain scores (*P* = 0.044). Both the QoR-15 scores and MoCA scores of patients in Groups A, B, and C were significantly higher than those of the Control Group (QoR-15: *P* = 0.001, < 0.001, < 0.001; MoCA: *P* = 0.004, < 0.001, < 0.001). There was no significant difference in QoR-15 between Group B and Group C (*P* = 0.054). No significant difference in hospitalization time was observed among the four groups (*P* = 0.256).

Anesthesia-related complications during the perioperative period: In the Group Control, there were four cases of intraoperative bradycardia and four cases of body movement or tongue injury. In Group A, there were two cases of bradycardia and one case of tongue injury. Group B had one case of stupor vigilans, while Group C had five cases of stupor vigilans (Table 3). All complications were promptly treated without causing severe consequences.

**TABLE 3:** Anesthesia-related complications during the perioperative period, *n* (%).

Complications	Group control	Group A	Group B	Group C	*P*
None	12 (60)	17 (85)	19 (95)	15 (75)	0.001
Bradycardia	4 (20)	2 (10)	0 (0)	0 (0)	–
Body movements, tongue injury	4 (20)	1 (5)	0 (0)	0 (0)	–
Stupor vigilans	0 (0)	0 (0)	1 (5)	5 (25)	–

## Discussion

This study first demonstrated that the combined administration of esketamine effectively reduces the requirement for propofol, remifentanil, and dexmedetomidine in spinal surgery with IONM. However, no significant impact was observed on operation duration, blood loss, intraoperative blood pressure, or hospitalization duration. Furthermore, the concurrent use of esketamine enhances short-term postoperative recovery quality and cognitive function. Notably, different doses of esketamine exhibit varying effects: while a dosage of 0.5 mg/kg/h provides superior postoperative analgesia, it prolongs recovery time; conversely, administering 0.3 mg/kg/h improves cognitive function recovery after hypotension occurrence. Employing esketamine also mitigates bradycardia and intraoperative body movement occurrences; however, excessive dosages may elevate the risk of stupor vigilans.

This study revealed that using esketamine, while ensuring anesthetic efficacy and circulatory stability, significantly reduces propofol, remifentanil, and dexmedetomidine requirements. Moreover, it diminishes the incidence of associated complications in line with previous research findings (12–16). Esketamine and propofol exhibit a complementary mechanism and anesthetic effect. Relevant literature has previously demonstrated that esketamine counteracts propofol-induced circulatory system inhibition through sympathetic effects, thereby mitigating the extent of blood pressure decline in patients (17, 18). The combined administration of esketamine and propofol enhances hemodynamic stability in surgical patients by ameliorating inflammatory response and stress during surgery.

Esketamine is frequently employed in combination with other medications to mitigate respiratory depression caused by opioids and high-dose sedatives while simultaneously improving postoperative recovery quality (14). A study investigating radical mastectomy for breast cancer revealed that esketamine not only enhances recovery quality and alleviates pain during the recovery period but also promotes the restoration of postoperative cognitive function without increasing adverse drug reaction risk. Similarly, another investigation found that combining dexmedetomidine with estazolam reduces terminal organ damage and pain perception while decreasing opioid requirements to enhance patient rehabilitation (19). Moreover, a study on thoracic surgery demonstrated that esketamine exhibits superior analgesic effects, improves negative emotions and sleep quality, and stabilizes intraoperative hemodynamics more effectively than dexmedetomidine. Additionally, it demonstrates better efficacy in preventing delirium and pain sensitization after anesthesia (20). In this study, despite the use of rocuronium during anesthesia induction, muscle relaxants were avoided throughout the operation to prevent potential interference with IONM signals. Consequently, the effects of esketamine and rocuronium on IONM may not be a significant concern.

In addition to its analgesic, sedative, and anesthetic effects, esketamine has also been found to possess the ability to prevent opioid-induced hyperalgesia (21). Yan et al. (22) propose that adding esketamine to sufentanil can enhance anesthesia quality and efficacy, reduce sufentanil dosage requirements, and mitigate potential adverse drug reactions. Saugel et al. (23) demonstrated that compared to sufentanil alone, patients receiving intravenous esketamine experienced improved hemodynamic stability during general anesthesia, required lower postoperative opioid consumption, and reported significantly reduced pain scores.

Additionally, it shortens postoperative recovery time and reduces adverse reactions such as respiratory depression and bradycardia. This anesthesia regimen yields superior outcomes in patients with hemodynamic instability (15, 24). Furthermore, esketamine possesses analgesic properties that effectively alleviate severe pain caused by propofol injection (15, 18). Propofol also improves neuropsychiatric response during the awakening period following esketamine administration, possibly due to its ability to reduce cerebral blood flow and intracranial pressure (25).

The present study also demonstrated the efficacy of esketamine in enhancing postoperative recovery quality and reducing the incidence of postoperative cognitive dysfunction. Previous investigations have consistently reported on the potential neuroprotective effects of esketamine, including its ability to prevent hyperalgesia, delirium, and postoperative cognitive dysfunction (13, 21, 24). When combined with propofol, esketamine effectively stabilizes cerebral blood flow velocity and reduces the incidence of postoperative cognitive dysfunction. This effect can be attributed to its neuroprotective properties (26). The results of a randomized controlled trial on elderly patients undergoing gastrointestinal surgery demonstrated that the intravenous administration of low-dose esketamine (0.125 mg/kg/h) significantly reduced the incidence of postoperative cognitive dysfunction (POCD) (16).

Esketamine exhibits a protective effect on the phenotype of stress perception induced by inflammation. The combined administration of propofol and esketamine mitigates patient drowsiness, anterograde amnesia, and a decline in short-term storage capacity (27). A study on laparoscopic cholecystectomy also demonstrates that the combination of dexmedetomidine and esketamine enhances surgical quality, alleviates postoperative agitation, and expedites cognitive function recovery in patients. This may be attributed to the direct inhibition of stress response through stimulation of the sympathetic nervous system by esketamine (28). In an investigation utilizing nuclear magnetic resonance imaging, hippocampal structures were scanned in healthy volunteers receiving esketamine. The findings reveal that esketamine augments plasticity within hippocampal neural structures, potentially elucidating the anatomical mechanism underlying its neuroprotective effects (29). These effects may be mediated through alterations in serum levels of S100 calcium-binding protein beta (S100β), neuron-specific enolase (NSE), as well as inflammatory factors such as IL-6, IL-8, and TNF-α (29).

The utilization of IONM imposes heightened demands on anesthesia management. When selecting an appropriate anesthesia technique, apart from minimizing interference with IONM data, it is imperative to consider minimizing risks associated with patient movement, consciousness alteration, and hemodynamic instability. Although low-concentration (0.5MAC) inhaled anesthetics are generally deemed suitable (30), complete avoidance of IONM data interference remains unattainable; hence, TIVA is frequently employed in clinical practice (31).

In this study, no significant effect of esketamine on the IONM signals was observed. Previous studies support that ketamine does not inhibit the IONM signal (32). Moreover, ketamine offers the advantage of postoperative pain reduction and minimization of adverse reactions (33). Therefore, when IONM surgery is required, low-dose ketamine emerges as a more suitable anesthetic choice (34). As an isomer of ketamine, esketamine further enhances its efficacy while reducing the incidence of adverse reactions (10, 11). Compared to ketamine, esketamine exhibits a significantly enhanced *in vivo* clearance rate, leading to a rapid decline in plasma concentration upon administration. Consequently, the utilization of esketamine results in mild respiratory depression for patients, minimal impact on systemic circulation, and reduced incidence of postoperative adverse reactions (35). Propofol combined with remifentanil is a commonly employed TIVA regimen, which effectively fulfills the sedation and analgesia requirements of patients. However, due to the absence of muscle relaxants, higher doses of intravenous anesthetics are necessary. Prolonged use of these drugs may elevate the risk of circulatory depression, postoperative hyperalgesia, and drug accumulation in patients, thereby hindering the achievement of stable anesthesia and causing interference during IONM (36). Dexmedetomidine, an adjunctive TIVA agent, can be utilized in surgeries involving IONM to reduce reliance on intravenous anesthetics (37, 38). Nevertheless, its usage may also impact IONM data (5, 38).

The findings of this study suggest that the administration of esketamine at a dosage of 0.3 mg/kg/h exhibits superior anesthetic efficacy and reduces postoperative complications. While increasing the dosage may enhance postoperative analgesia, it does not contribute to overall effectiveness; instead, it prolongs postoperative recovery time and increases the likelihood of stiffness (although it is temporary and reversible, it increases the patient’s unpleasant experience). Compared to a dosage of 0.5 mg/kg/h, there is no significant difference in promoting postoperative rehabilitation and reducing postoperative cognitive dysfunction with a dosage of 0.3 mg/kg/h, indicating that higher doses are unnecessary. Previous studies have utilized esketamine dosages similar to or lower than those employed in this study (12, 13, 15, 16, 18, 20, 25, 26). Considering the requirement for IONM, slightly higher drug dosages may be necessary when muscle relaxants are not used, which is both reasonable and feasible.

However, it is essential to note that esketamine elicits sympathetic nerve stimulation and inhibits catecholamine reuptake, leading to increased heart rate, cardiac output, heightened blood pressure, and augmented cerebral blood flow. Simultaneously, it also induces an increase in intracranial pressure and intraocular pressure. Therefore, caution should be exercised when administering this medication to patients with hypertension, eclampsia, hyperthyroidism, ischemic heart disease, mental illness, epilepsy, glaucoma, or a history of cerebrovascular accidents.

The present study still needs to exhibit certain limitations. Although there was no significant difference in preoperative HR and MAP among the groups, there was still a robust trend, which may limit the generalizability of our findings. Future studies with larger sample sizes are needed to further validate our results. Due to the limitation of disease types, the age range of the included patients was too wide (18–75 years), and stratified subgroup analysis was lacking to analyze the possible impact brought by the age factor. Additionally, the duration of follow-up in our study was relatively short, and longer-term outcomes should be investigated to fully understand the impact of esketamine and propofol combination therapy on postoperative recovery and cognitive function.

In conclusion, esketamine can be safely and effectively used in spinal surgery under IONM, thereby reducing the reliance on intravenous anesthetic agents. Moreover, the dose of 0.3 mg/kg/h esketamine is better regarding the anesthetic effect and control of postoperative complications.

## Data Availability

The original contributions presented in this study are included in this article/supplementary material, further inquiries can be directed to the corresponding author.
